# Primary anorectal melanoma: report of two cases

**DOI:** 10.1093/jscr/rjaf376

**Published:** 2025-06-06

**Authors:** Ever Frank Lopez-Cutipa, Lizbeth Katherine Quintero-Aquino, Cesar Torres-Mattos

**Affiliations:** Coloproctology Surgery, Guillermo Almenara National Hospital, Av. Grau 800, La Victoria, Lima, 15018, Peru; Surgical Pathology and Necropsies, Guillermo Almenara National Hospital, Av. Grau 800, La Victoria, Lima, 15018, Peru; Medical Oncology, Guillermo Almenara National Hospital, Av. Grau 800, La Victoria, Lima, 15018, Peru

**Keywords:** melanoma, anal neoplasms, anorectal diseases, amelanotic melanoma, immunotherapy

## Abstract

Anorectal melanoma is a rare and aggressive neoplasm with a poor prognosis; the overall survival rate at 5 years is around 20%. We report two clinical cases involving patients who experienced pain and bleeding associated with an anorectal mass. Histopathological studies confirmed the diagnosis of melanoma. The first case presented with localized disease characterized by a black, pedunculated lesion with irreducible prolapse managed with wide local excision. In the second case, the patient had a fixed, pink anal lesion accompanied by multiple inguinal and retroperitoneal lymphadenopathies. Due to the metastatic nature of the disease, immunotherapy was administered. Both patients remain alive after 1 year of follow-up.

## Introduction

Anorectal melanoma (ARM) is a rare disease with a poor prognosis. Its incidence ranges from 0.3 to 1 case per million people, and its frequency has increased in recent years [1]. ARM accounts for approximately 0.05% of colorectal malignancies and 1% to 4% of anal canal malignancies [2]. Common symptoms include rectal bleeding, anorectal pain, or the sensation of a mass, which are often mistaken for benign conditions such as hemorrhoids or polyps, leading to delays in diagnosis [1]. The diagnosis presents significant challenges due to the anatomical location of the lesions, nonspecific clinical symptoms, variable histopathological morphology, and the fact that up to 40% of lesions may show amelanotic characteristics [3, 4]. For nonmetastatic cases, surgery is the main treatment option [2]. Two surgical strategies are available: radical surgery (abdominoperineal resection, APR) and wide local excision (WLE). No statistically significant difference in survival outcomes has been found between these strategies [1, 2]. The five-year overall survival rate is estimated to range between 20% and 22%. Median survival is approximately 24 months for stage I (local involvement), 17 months for stage II (nodal metastasis), and 8 months for stage III (distant metastasis) [3].

We present two illustrative cases of ARM and review relevant literature.

## Case series

### Case 1

**Figure 1:**
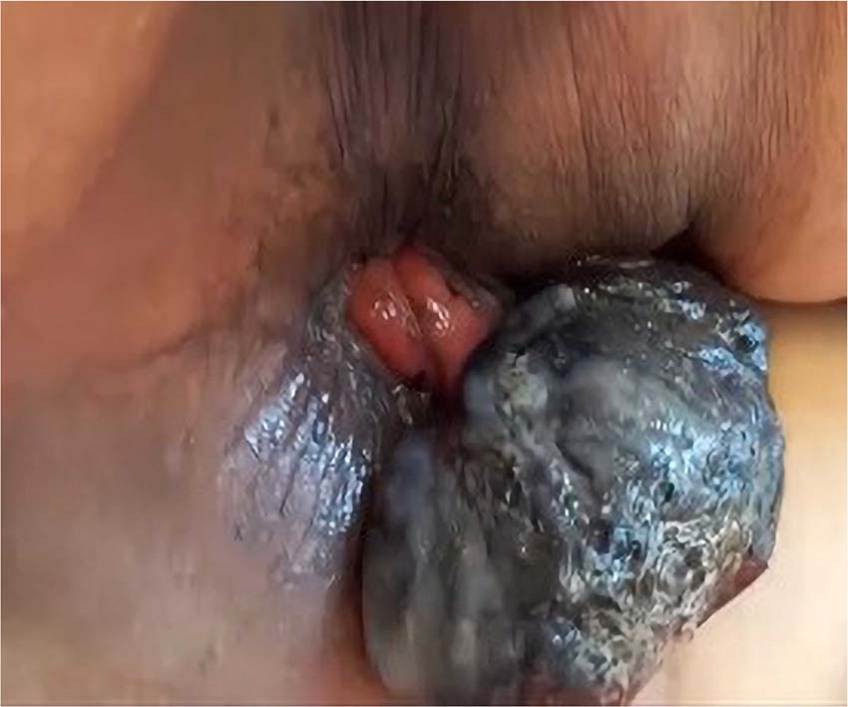
Clinical image of Case 1 showing a black, pedunculated anorectal tumor arising from the pectinate line, presenting as an irreducible prolapsed mass.

**Figure 2:**
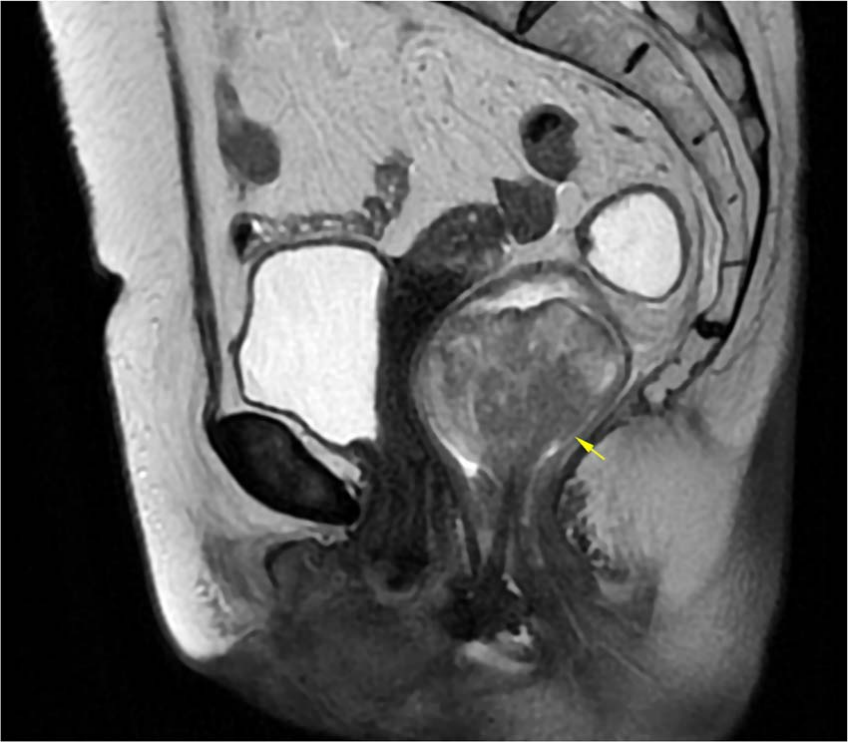
MRI of Case 1 shows a well-defined rectal tumor without regional lymph node involvement. The lesion demonstrates high signal intensity on sagittal T2-weighted imaging (indicated by the arrow).

A 69-year-old woman with no significant medical history presented with rectal bleeding and a manually reducible prolapsed tumor for the past 3 months. A colonoscopy revealed a black, pedunculated tumor with a base at 6 o’clock, located along the pectineal line, measuring 5 cm in diameter, with erosion and signs of recent bleeding. Biopsy confirmed malignant melanoma. Before admission, the patient experienced anal pain and observed that the tumor had become irreducible. On examination, the tumor was pedunculated, irreducible, mobile, soft in consistency, black in color, and emitted a putrid odor (Fig. 1). Blood tests showed hemoglobin at 9.4 g/dl and a C-reactive protein level of 34.9 mg/l. A magnetic resonance imaging (MRI) study showed a well-defined rectal mass measuring 53 × 47 × 44 mm, with no regional lymph node involvement (Fig. 2). The computerized tomography (CT) scan showed no distant metastases. The multidisciplinary committee decided on WLE. Postoperative recovery was uneventful, and the patient was discharged after 48 h.

**Figure 3:**
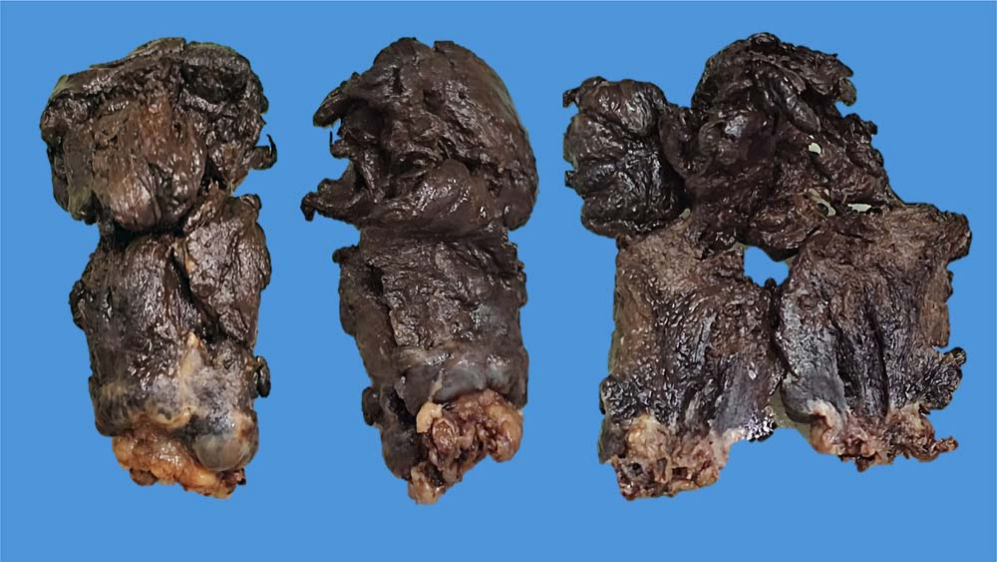
Macroscopic image of the surgical specimen from Case 1 depicting a blackish, pedunculated, polypoid lesion. The opened specimen shows anal mucosa at its base.

**Figure 4:**
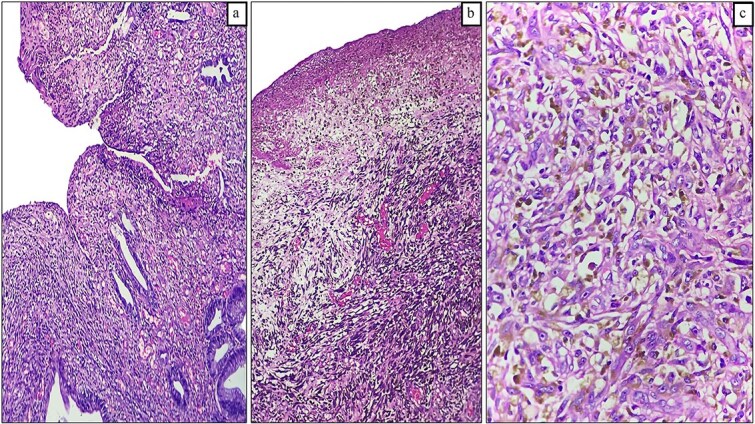
Histopathological findings of Case 1. (a) Panoramic image of anorectal melanoma. (b) Ulcerated area with neoplastic cells beneath the surface (hematoxylin and eosin [H&E] staining, 100×). (c) Epithelioid neoplastic cells with melanin pigment in the cytoplasm (H&E staining, 400×).

Pathology revealed a pedunculated polypoid tumor measuring 55 mm (Fig. 3), consistent with an ulcerated malignant melanoma exhibiting deep invasion greater than 4 mm, radial lentiginous growth, and no evidence of lymphovascular invasion. Tumor cells exhibited significant pigmentation and a mitotic rate of 5 per 10 high-power fields. Surgical margins were tumor-free (Fig. 4). Immunohistochemistry (IHC) was positive for Melan A, S100, P53, and CD117. One year after surgery, the patient remains free of recurrence and attends follow-ups every 4 months.

### Case 2

**Figure 5:**
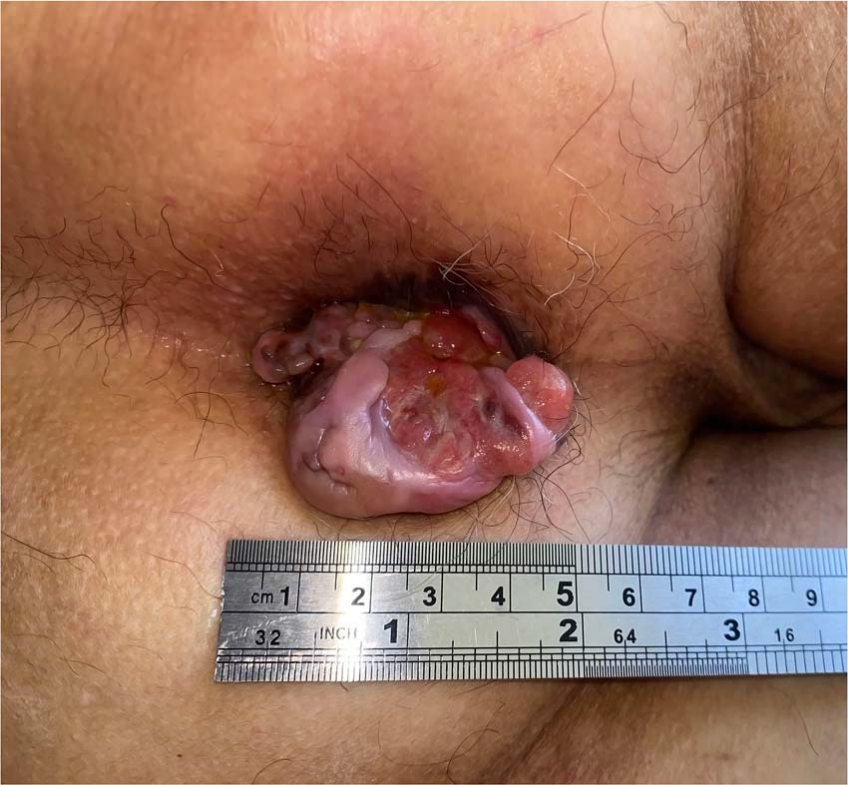
Clinical image of Case 2 showing a pink tumor in the anal region with superficial ulceration.

**Figure 6:**
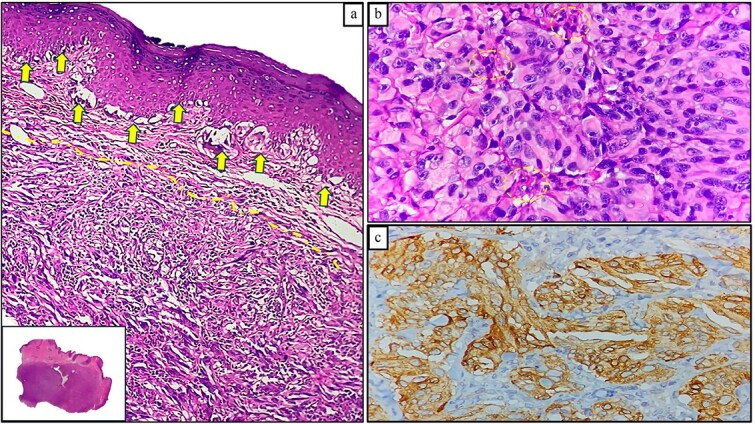
Histopathological findings of Case 2. (a) ARM exhibiting radial (solid arrows) and vertical (dotted lines) growth patterns; lower inset shows a panoramic view (H&E staining, 100×). (b) Malignant melanocytes with cytologic atypia and epithelioid morphology, displaying minimal melanin pigment (dotted circles; H&E staining, 400×). (c) Positive Melan-a expression in neoplastic cells (immunohistochemical staining, 100×).

**Figure 7:**
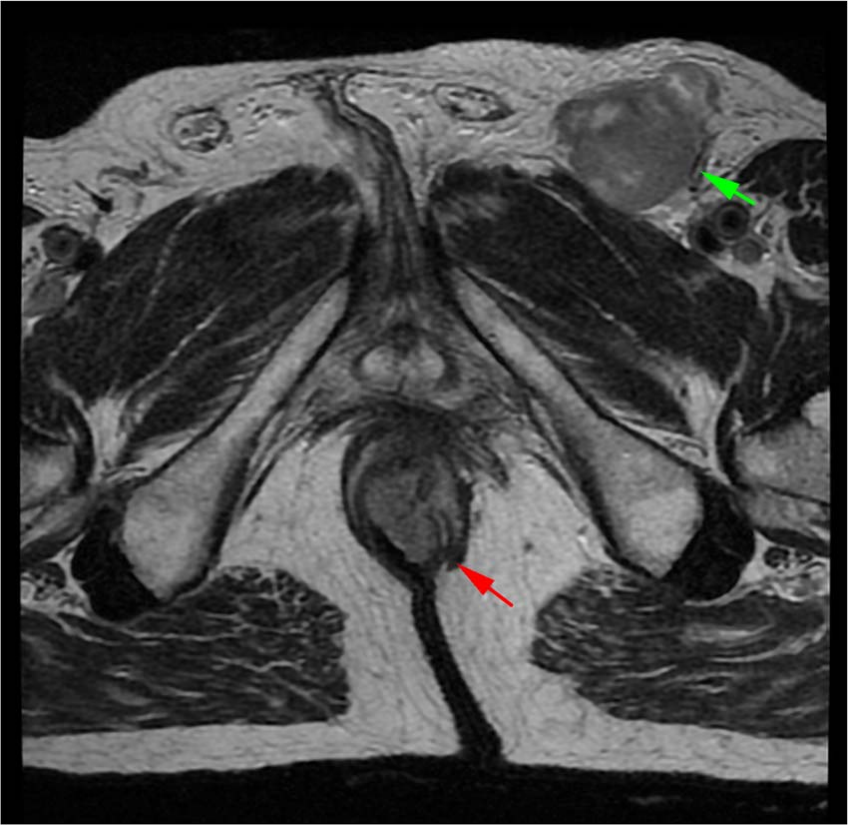
MRI of Case 2 shows an infiltrating anal tumor involving the internal and external sphincters, as well as perianal adipose tissue (lower arrow), with associated inguinal lymphadenopathy (upper arrow). The tumor demonstrates high signal intensity on axial T2-weighted imaging.

A 71-year-old man reported pain and bleeding associated with an anal tumor over 6 months. Colonoscopy showed no colorectal lesions, but an anal tumor was identified in the anterior region. It was fixed, firm, and approximately 4 cm in diameter, pink, with signs of superficial erosion and recent bleeding (Fig. 5). A biopsy confirmed the presence of ulcerated malignant melanoma with deep invasion greater than 4 mm and mild pigmentation. IHC was positive for Melan A, S100, and CD117 (Fig. 6). Blood tests were within normal limits. MRI revealed a lesion in the lower two-thirds of the anal canal (35 × 24 × 35 mm), located between the 12 and 1 o’clock positions, infiltrating the external sphincter and perianal fat (Fig. 7). Additionally, multiple metastatic lymphadenopathies were identified in the left (44 × 34 mm) and right (10 × 10 mm) inguinal regions, as well as in the internal and external iliac chains, which were histologically confirmed. The CT scan supported these findings, indicating the presence of retroperitoneal metastatic lymphadenopathies. The multidisciplinary committee recommended immunotherapy with nivolumab (240 mg IV every 14 days). After 12 treatment cycles, the patient showed good tolerance and adherence, with no evidence of disease progression.

## Discussion

ARM belongs to the group of mucosal melanomas (MM), a rare subtype that constitutes approximately 1% of all melanomas. Within this category, ARM accounts for about 24% of cases [3]. ARM pathogenesis is unclear. While ultraviolet radiation is the primary risk factor for the development of cutaneous melanoma (CM), it seemingly does not contribute to the genesis of ARM [4]. The concept of different pathogenesis between CM and MM is supported by the demonstrated variation in their mutational profiles. BRAF (>50%) and NRAS (15%–28%) mutations are common in CM, but less so in ARM (9% and 4%, respectively), where mutations in KIT, SF3B1, NRG1, and NF1 are more frequent [5, 6].

The mean age at presentation is approximately 68 years, with a slight predominance of females [1]. This is consistent with the ages of the cases presented. Symptoms include bleeding in 84.9% of cases and anorectal pain in 68.7% [7]; however, changes in bowel habits, tenesmus, palpable anal masses, and pruritus are also observed. Both cases in this study presented with bleeding, pain, and palpable masses: hyperpigmented in one case and pink, amelanotic in the other; the latter condition has been reported in approximately 40% of patients with ARM [4].

Tumors may arise in the rectum (above the dentate line), directly at the dentate line, or in the anus (below it). The importance of this distinction remains debatable. Retrospective studies did not show a statistically significant difference in survival when comparing locations [5]. However, a recent analysis based on the epidemiologic surveillance program of the National Cancer Institute of the United States examined 765 patients with ARM, finding that rectal melanoma has a worse disease-specific survival than anal melanoma [8]. In our cases, the lesions were at the pectineal line and anus, with the anal lesion showing more aggressive behavior.

Primary ARM diagnosis requires an *in situ* component, a radial growth phase of focal melanocytosis, and no history of CM [4]. Tumor cells may be epithelioid, spindle, or mixed, exhibiting polymorphism and anaplasia. Depth of invasion (greater than 4 mm) and lymphovascular invasion are considered histopathological factors indicating a poor prognosis [9]. IHC markers consist of S100, HMB-45, Melan A, vimentin, and CD117.

There is no classification from the American Joint Committee on Cancer for MAR. However, some authors utilize a classification based on three stages: (i) local disease, (ii) local disease with regional lymph nodes, and (iii) distant metastatic disease [3, 4]. Surgery is the mainstay of treatment for nonmetastatic disease [2], with commonly used surgical approaches including WLE and APR [1–5]. Many studies support WLE due to the uncertain outcomes associated with APR, the reduced quality of life associated with radical surgery, and the risk of ending up with a permanent colostomy [1]. While it has been suggested that APR might enhance local recurrence rates compared to WLE [10, 11], other studies have reported no difference in these rates [1]. Due to the low prevalence of the disease, there are currently no randomized clinical trials comparing WLE and APR. However, retrospective studies and meta-analyses have not shown improved overall or disease-free survival with APR compared to WLE [1–8]. Therefore, in the absence of a clear survival benefit, the current trend is to perform WLE [5, 12, 13] and use APR selectively for large, advanced, resectable, nonmetastatic tumors that are unsuitable for WLE [2]. We opted for WLE in the first case because it was a mobile and pedunculated lesion, successfully achieving negative margins and functional preservation.

Adjuvant therapy lacks consensus. Previous studies indicate that administering radiotherapy after anal sphincter-sparing surgery can improve local disease control. However, the benefits should be carefully considered against potential adverse events and the absence of evidence suggesting that it improves survival rates [2]. Single-agent adjuvant chemotherapy is well tolerated but has a response rate of less than 20%. In contrast, multi-agent chemotherapy can boost response rates, although it comes with greater toxicity and no significant improvement in survival [14]. In a study of 450 patients with nonmetastatic ARM, 64% were treated with surgery alone, and radiotherapy was the most common adjuvant therapy [2]. Our first case received surgery only and remains recurrence-free at 12 months.

The role of immunotherapy with PD-1 immune checkpoint blockade and BRAF-targeted therapy is well-established for unresectable stage III and metastatic stage IV CM [15]. In relation to MM, evidence suggests a reduced efficacy of immunotherapy compared to MC, likely due to their differing mutational profiles [1, 15]. Nevertheless, some cases of MM exhibit prolonged responses to this therapy [15]. A clinical trial involving 889 patients, with 10% having MM, evaluated the efficacy and safety of nivolumab, both alone and in combination with ipilimumab. It showed a median progression-free survival of approximately 3 months for monotherapy and 6 months for combination therapy in patients with unresectable or metastatic MM [3]. In this context, several retrospective studies suggest that the use of immunotherapy is beneficial for the survival of patients with metastatic ARM [14]. Our second patient received nivolumab and achieved 6 months of progression-free survival.

ARM has a poor prognosis, exhibiting a 5-year overall survival rate of 20% to 22% [3]. At diagnosis, 24%–40% have distant metastases [9, 11], and 34% have regional spread [9]. A retrospective study of 38 patients with stage I ARM treated with curative surgical resection reported that 58% of cases experienced recurrence. Of these, 50% were attributed to distant metastases, most commonly affecting the liver and lungs [12]. These findings suggest that survival from ARM is influenced by metastatic disease, which occurs in the majority of patients, regardless of the surgery performed on the primary tumor [1].

Our report provides insight into ARM presentation and management but is limited by a short follow-up period and a lack of molecular analysis. Further studies are necessary to clarify resistance mechanisms and identify clinical and molecular predictors, which could lead to more effective therapy strategies.

## Conclusion

ARM carries a poor prognosis due to frequent metastases at diagnosis or after surgery. Surgery remains the preferred treatment; radical resection should be reserved for tumors that cannot be resected locally. Immunotherapy should be considered within a multimodal strategy.
